# Influence of Ethylene Signaling in the Crosstalk Between Fe, S, and P Deficiency Responses in *Arabidopsis thaliana*

**DOI:** 10.3389/fpls.2021.643585

**Published:** 2021-03-30

**Authors:** María José García, Macarena Angulo, Carlos García, Carlos Lucena, Esteban Alcántara, Rafael Pérez-Vicente, Francisco Javier Romera

**Affiliations:** ^1^Department of Botany, Ecology and Plant Physiology, Edificio Celestino Mutis, Campus de Rabanales CeiA3, Universidad de Córdoba, Córdoba, Spain; ^2^Department of Agronomy (DAUCO-María de Maeztu Unit of Excellence), Edificio Celestino Mutis, Campus de Rabanales CeiA3, Universidad de Córdoba, Córdoba, Spain

**Keywords:** iron, ethylene, sulfur, phosphatase, reductase, phosphorus

## Abstract

To cope with P, S, or Fe deficiency, dicot plants, like *Arabidopsis*, develop several responses (mainly in their roots) aimed to facilitate the mobilization and uptake of the deficient nutrient. Within these responses are the modification of root morphology, an increased number of transporters, augmented synthesis-release of nutrient solubilizing compounds and the enhancement of some enzymatic activities, like ferric reductase activity (FRA) or phosphatase activity (PA). Once a nutrient has been acquired in enough quantity, these responses should be switched off to minimize energy costs and toxicity. This implies that they are tightly regulated. Although the responses to each deficiency are induced in a rather specific manner, crosstalk between them is frequent and in such a way that P, S, or Fe deficiency can induce responses related to the other two nutrients. The regulation of the responses is not totally known but some hormones and signaling substances have been involved, either as activators [ethylene (ET), auxin, nitric oxide (NO)], or repressors [cytokinins (CKs)]. The plant hormone ET is involved in the regulation of responses to P, S, or Fe deficiency, and this could partly explain the crosstalk between them. In spite of these crosslinks, it can be hypothesized that, to confer the maximum specificity to the responses of each deficiency, ET should act in conjunction with other signals and/or through different transduction pathways. To study this latter possibility, several responses to P, S, or Fe deficiency have been studied in the *Arabidopis* wild-type cultivar (WT) Columbia and in some of its ethylene signaling mutants (*ctr1, ein2-1, ein3eil1*) subjected to the three deficiencies. Results show that key elements of the ET transduction pathway, like CTR1, EIN2, and EIN3/EIL1, can play a role in the crosstalk among nutrient deficiency responses.

## Introduction

Phosphorus (P), sulfur (S), and iron (Fe) are essential mineral elements for plants (Takahashi et al., 2011; Briat et al., 2015a,b; Lucena et al., 2015). P represents a building block for vital organic molecules such as DNA, RNA, ATP, and phospholipids (Ajmera et al., 2019; Crombez et al., 2019), and S and Fe, among other functions, are needed for many proteins of the photosynthetic and respiratory chains (Couturier et al., 2013; Vigani and Briat, 2016; Mendoza-Cózatl et al., 2019). The main sources of P, S, and Fe for plants are phosphate, sulfate, and Fe ions (Fe^2+^ for dicot plants), which are absorbed through specific transporters (Maruyama-Nakashita et al., 2006; Kobayashi and Nishizawa, 2012; Briat et al., 2015b; Lucena et al., 2015; Wawrzynska and Sirko, 2016; Ajmera et al., 2019; Crombez et al., 2019). Phosphate, sulfate, and Fe ions have low availability in some soil conditions (i.e., Fe ions in calcareous soils or phosphate in both calcareous and acid soils) in such a way that P, S, and Fe deficiencies have become a global threat for agricultural food production (Briat et al., 2015a,b; Lucena et al., 2015, 2018; Zuchi et al., 2015; Ajmera et al., 2019; Crombez et al., 2019; Venuti et al., 2019; Siddiqui et al., 2020).

To cope with P, S, or Fe deficiency, dicot plants develop several physiological and morphological responses (mainly in their roots) aimed at facilitating the mobilization and acquisition of these nutrients. Among these responses are the enhanced expression of specific transporters and the alteration of the root system architecture (Maruyama-Nakashita et al., 2006; Takahashi et al., 2011; Zhang et al., 2014; Briat et al., 2015b; García et al., 2015; Lucena et al., 2015; Ajmera et al., 2019; Crombez et al., 2019; Venuti et al., 2019). In *Arabidopsis*, the main phosphate transporters implicated in its acquisition from the medium are PHT1;1 and PHT1;4 (also named PT1 and PT2; Nagarajan and Smith, 2012; Lucena et al., 2018, 2019; Crombez et al., 2019); the ones for sulfate are SULTR1;1 and SULTR1;2 (Maruyama-Nakashita et al., 2006; Takahashi et al., 2011; Yamaguchi et al., 2020), and the one for Fe^2+^ is IRT1 (Kobayashi and Nishizawa, 2012; Lucena et al., 2015). Nutrient deficiencies also alter the expression of internal transporters, like PHT1;5, which plays a critical role in mobilizing phosphate from source to sink organs (Nagarajan et al., 2011; Nagarajan and Smith, 2012; Zhang et al., 2014).

Both Fe and P deficiencies can induce other responses, like the acidification of the rhizosphere; the increased synthesis and release of organic acids and phenolics to the rhizosphere; and the development of subapical root hairs (Kobayashi and Nishizawa, 2012; Correia et al., 2014; Wang et al., 2014; Zhang et al., 2014; García et al., 2015; Lucena et al., 2015, 2018, 2019; Neumann, 2016; Tsai and Schmidt, 2017; Venuti et al., 2019). Particularly, Fe deficiency can induce an enhanced ferric reductase activity (FRA), due to increased expression of the *FRO2* gene (Kobayashi and Nishizawa, 2012; Lucena et al., 2015), and P deficiency an enhanced phosphatase activity (PA), due to increased expression of *PAP* genes, like *PAP7* or *PAP17* (also named *ACP5*; Lei et al., 2011; Zhang et al., 2014; Lucena et al., 2018, 2019; Venuti et al., 2019).

In recent years, considerable advances have been achieved in the identification of genes related to different responses in *Arabidopsis* and in other plant species and in the master transcription factors (TFs) regulating them. In relation to P deficiency, the master TF is PHR1, a MYB TF which interacts with other related TFs, like PHL1, to activate the expression of P starvation genes, like *PHT1;1, PHT1;4*, and *PAP17* (Rubio et al., 2001; Bustos et al., 2010; Nagarajan and Smith, 2012; Briat et al., 2015b; Sun et al., 2016). In relation to Fe deficiency, the master TF is FIT, a bHLH TF which interacts with other related bHLH TFs, like bHLH38 and bHLH39, to activate the expression of Fe starvation genes, like *FRO2* and *IRT1* (Kobayashi and Nishizawa, 2012; Lucena et al., 2015; Gao et al., 2019; Schwarz and Bauer, 2020). Finally, in relation to S deficiency, the master TF is SLIM1, an ethylene insensitive 3-like (EIL3) family TF which could interact with other TFs to activate the expression of S starvation genes, like *SULTR1;1* and *SULTR1;2* (Maruyama-Nakashita et al., 2006; Takahashi et al., 2011; Wawrzynska et al., 2015; Wawrzynska and Sirko, 2016, 2020; Yamaguchi et al., 2020). These master TFs could be regulated transcriptionally and post-transcriptionally. In the case of *FIT*, its transcriptional (increased expression in Fe-deficient roots) and post-transcriptional regulation is clear (Lingam et al., 2011; Yang et al., 2014; Lucena et al., 2015; Gao et al., 2019; Wu and Ling, 2019; Schwarz and Bauer, 2020). However, the transcriptional regulation of *PHR1* and *SLIM1* is less clear and presents some controversy. While some authors have found *PHR1* upregulation under P deficiency (Huang et al., 2018), others did not (Rubio et al., 2001; Sega and Pacak, 2019 and references therein). For *SLIM1*, there are no data supporting its upregulation under S deficiency (Maruyama-Nakashita et al., 2006; Wawrzynska and Sirko, 2016).

The responses to nutrient deficiencies share two important characteristics. First, they are induced transiently in such a way that, once the nutrient has been acquired in enough quantity, the responses are switched off to minimize energy costs and toxicity. In this sense, it should be noted that Fe^2+^ and S^2−^ are highly reactive and potentially toxic (Mendoza-Cózatl et al., 2019). Second, although the responses to each deficiency are rather specific, coordination and crosstalk between them do exist. This is reflected in many results showing that a nutrient deficiency can alter the content and expression of genes related to other nutrient(s). Fe deficiency can induce the expression of sulfate and phosphate acquisition genes while P or S deficiency can induce the expression of Fe acquisition genes and its accumulation (Ward et al., 2008; García et al., 2010; Paolacci et al., 2014; Briat et al., 2015b and references therein; Lucena et al., 2015, 2018 and references therein, 2019; Zuchi et al., 2015; Venuti et al., 2019). Some P-related genes, like *PAP17* and *PHT1;4*, and PA, are induced under Fe deficiency (García et al., 2010; Lucena et al., 2019; Park et al., 2019). P deficiency can also induce the expression of sulfate transporter genes (Rouached et al., 2011).

One possible reason for the crosstalk between P, S, and Fe deficiency could be the participation of the same hormones and signaling molecules, like ethylene (ET), auxin, and nitric oxide (NO), in the activation of the responses to the different deficiencies (Lucena et al., 2006, 2015, 2018, 2019; Romera et al., 2011, 2016, 2017, 2021; Nagarajan and Smith, 2012; Zhang et al., 2014; García et al., 2015; Song and Liu, 2015; Wawrzynska et al., 2015; Koprivova and Kopriva, 2016; Neumann, 2016; Liu et al., 2017; Huang et al., 2018; Buet et al., 2019; Galatro et al., 2020; Siddiqui et al., 2020). In relation to ET, its involvement in the regulation of P, S, and Fe deficiency responses is supported by many experimental results. At first, a higher ET production has been detected in P-, S-, and Fe-deficient roots, associated with the upregulation of ET synthesis and signaling genes (Romera and Alcántara, 2004; Zuchi et al., 2009; Nagarajan and Smith, 2012; García et al., 2015; Lucena et al., 2015; Moniuszko, 2015; Song and Liu, 2015; Wawrzynska et al., 2015; Romera et al., 2016, 2017, 2021; Li and Lan, 2017). On the other hand, ET has been involved in the upregulation of key Fe acquisition genes, like *FIT, FRO2*, and *IRT1* (Lucena et al., 2006; García et al., 2010), and P acquisition genes, like *PHT1;1, PHT1;4*, and *PAP17* (Lei et al., 2011). Finally, in relation to S nutrition, several S-responsive genes, like *SULTR1;1*, are upregulated by S limitation more significantly in wild-type (WT) *Arabidopsis* than in *slim1* mutants (Maruyama-Nakashita et al., 2006). *SLIM1* was identified as an allele of EIL3 (see above; Maruyama-Nakashita et al., 2006), possibly related to ET signaling (Wawrzynska et al., 2015; Wawrzynska and Sirko, 2016, 2020).

ET's mode of action is not fully understood, but a linear canonical signaling pathway has been proposed in *Arabidopsis* (Shakeel et al., 2013; Wang et al., 2013; Dubois et al., 2018; Binder, 2020):

$$\begin{array}{l} \text{ET}-∥\text{ETreceptors}\to \text{CTR}1-∥\text{EIN}2\to \text{EIN}3/\\ \text{ EILs}\to \text{ERFs}\to \text{ET responses} \end{array}$$

In this signaling pathway, EIN3/EILs and ERFs are TFs (for more details, see Shakeel et al., 2013; Wang et al., 2013; Lucena et al., 2015; Dubois et al., 2018; Binder, 2020). According to this pathway, *ctr1* mutants present constitutive responses to ET while *ein2, ein3*, and *eil*s are insensitive to ET (Wang et al., 2013). In recent years, several components of this signaling pathway have been implicated in the regulation of the master TFs controlling P, S, and Fe deficiency responses. In Fe deficiency responses, it has been shown that EIN3 and EIL1 interact with MED16 (mediator) to form a complex involved in the transcription of FIT (Yang et al., 2014). Moreover, Lingam et al. (2011) found that EIN3 and EIL1 also participate in the post-transcriptional regulation of FIT. In P deficiency responses, EIN3/EIL1 have been implicated in PHR1 expression (Liu et al., 2017) while in S deficiency responses, EIN3 has been proposed to negatively interact with SLIM1 for the upregulation of S acquisition genes (Wawrzynska and Sirko, 2016).

In Fe and P deficiency responses, the activating effect of ET is dependent on the Fe or P status of the plants, which suggests the involvement of Fe- and P-related repressive signals that would counteract ET action (García et al., 2011, 2013, 2015, 2018; Lei et al., 2011; Romera et al., 2017, 2021). In agreement with this affirmation, neither the *Arabidopsis* ET constitutive mutant *ctr1* nor the *Arabidopsis* ET overproducer mutant *eto* present full constitutive activation of P and Fe acquisition genes when grown under nutrient sufficiency (Lei et al., 2011; García et al., 2014, 2015). An essential question is whether ET regulates all the P, S, and Fe deficiency responses through the same transduction pathway or not. The results obtained with different ET signaling mutants suggest that it does not. For example, the upregulation of the *PHT1;4* (*PT2*) gene, induced under P deficiency, is drastically impaired in the *Arabidopsis* ET-insensitive *ein2* mutant (Lei et al., 2011) while the one of the *IRT1* gene, induced under Fe deficiency, is not (García et al., 2010; Angulo et al., 2021).

In this work, we have examined different P, S, and Fe physiological responses in *Arabidopsis* WT Columbia and some of its ET signaling mutants (*ctr1, ein2-1*, and *ein3eil1*) subjected to the three deficiencies. The objective has been to gain insight into the role of ET signaling components in the crosstalk between the three deficiencies. The election of the mutants is based on several reasons. At first, CTR1 and EIN2 are key components of the ET canonical signaling pathway and EIN3/EIL1 have been involved in the activation of the master TFs controlling the regulation of Fe, P, and S acquisition genes (see above). Furthermore, while *ctr1* mutants show constitutive responses to ET, *ein2* and *ein3eil1* are insensitive to ET, which allow to contrast their possible activating or deactivating role on the responses.

## Materials and Methods

### Plant Materials, Growth Conditions, and Treatments

To analyze the effects of Fe, P, or S deficiency on the induction of Fe-, P-, and S-related responses, we used WT *Arabidopsis* (*Arabidopsis thaliana* (L.) Heynh ecotype Columbia) plants. In addition, we used the *Arabidopsis ctr1* mutant that shows constitutive upregulation of ET responses (Guo and Ecker, 2003; Huang et al., 2003) and the *ein2-1* and *ein3eil1* mutants that show insensitivity to ET (Shakeel et al., 2013; Wang et al., 2013; Dubois et al., 2018; Binder, 2020; all mutants were obtained from the European Arabidopsis Stock Center). *Arabidopsis* plants were grown under controlled conditions as previously described (Lucena et al., 2006, 2007). Briefly, seeds were germinated in black peat and, when appropriate, seedlings were transferred to individual containers (of 70 ml volume) with complete nutrient solution continuously aerated. The nutrient solution had the following composition: macronutrients: 2 mM Ca(NO_3_)_2_, 0.75 mM K_2_SO_4_, 0.65 mM MgSO_4_, 0.5 mM KH_2_PO_4_, and micronutrients: 50 μM KCl, 10 μM H_3_BO_3_, 1 μM MnSO_4_, 0.5 μM CuSO_4_, 0.5 μM ZnSO4, 0.05 μM (NH_4_)_6_Mo_7_O_24_, and 20 μM Fe-EDDHA. Plants were grown in a growth chamber at 22°C day/20°C night temperatures, with relative humidity between 50 and 70%, and an 8-h photoperiod (to postpone flowering) at a photosynthetic irradiance of 300 μmol m^−2^ s^−1^ provided by fluorescent tubes (Sylvania Cool White VHO).

When plants were ~45 days old, they were directly transferred, without washing, from this complete nutrient solution to the different treatments. The treatments imposed were: control: complete nutrient solution with 40 μM Fe-EDDHA; –Fe: nutrient solution without Fe; –P: nutrient solution without P (0.5 mM KH_2_PO_4_ was not added to the nutrient solution; instead, 0.5 mM KCl was added); –S: nutrient solution without S (only 2 μM S from the micronutrients added; 0.75 mM K_2_SO_4_ and 0.65 mM MgSO_4_ were not added to the nutrient solution; instead, 1.5 mM KCl and 0.65 mM Mg(NO_3_)_2_ were added). After 2, 4, 6, and 8 days of the treatments, root ferric reductase activity (FRA) and acid phosphatase activity (PA) were determined in plants from the four treatments, as previously described (Lucena et al., 2006, 2019; see also below). After FRA determination, roots were collected and kept at −80°C for subsequent analysis of mRNA levels. Each experiment was repeated at least twice, and representative results are presented.

### Acid Phosphatase Activity

It was determined as previously described (Zakhleniuk et al., 2001). Briefly, roots of intact plants were placed in Petri dishes containing a solution with 5-bromo-4-chloro-3′-indolyphosphate *p*-toluidine salt (BCIP) 0.01% (*w*/*v*) for 4 h. Blue color of roots is higher with increased PA. After 4 h, photographs of roots were taken with a stereoscopic microscope.

### Root Ferric Reductase Activity

It was determined as previously described (Lucena et al., 2006, 2007). Briefly, intact plants were placed in a Fe(III) reduction assay solution containing Fe(III)-EDTA and ferrozine for 60 min. The FRA was determined spectrophotometrically by measuring the absorbance (562 nm) of the Fe(II)-ferrozine complex and using an extinction coefficient of 29,800 M^−1^ cm^−1^. After the reduction assay, roots were excised and weighed, and the results were expressed on a root fresh weight basis. Data are given as means of six replicates.

### qRT-PCR Analysis

Roots were ground to a fine powder with a mortar and pestle in liquid nitrogen. Total RNA was extracted using the Tri Reagent solution (Molecular Research Center, Inc. Cincinnati, OH, USA) according to the manufacturer's instructions. M-MLV reverse transcriptase (Promega, Madison, WI, USA) was used to generate cDNA from 3 μg of DNase-treated root RNA as the template and random hexamers as the primers.

The study of gene expression by qRT-PCR was performed by using a qRT-PCR Bio-Rad CFX connect thermal cycler and the following amplification profile: initial denaturation and polymerase activation (95°C for 3 min), amplification, and quantification repeated 40 times (90°C for 10 s, 57°C for 15 s, and 72°C for 30 s), and a final melting curve stage of 65 to 95°C with increment of 0.5°C for 5 s, to ensure the absence of primer dimer or nonspecific amplification products. PCR reactions were set up in 20 μl of SYBR Green Bio-RAD PCR Master Mix, following the manufacturer's instructions. Controls containing water instead of cDNA were included to check for contamination in the reaction components. Gene-specific primers used are listed in Table 1. Oligonucleotides in García et al. (2018) were used to amplify *FRO2, IRT1*, and *FIT* cDNA, and those in Lucena et al. (2019) and Maruyama-Nakashita et al. (2006) to amplify *PAP17* and *SULTR1;1*, respectively. Oligonucleotides used to amplify *PHT1;5, PHR1* and *SLIM1/EIL3* were designed by using the Primer-BLAST software from the NCBI site. Standard dilution curves were performed for each primer pair to confirm appropriate efficiency of amplification (*E* = 100 ± 10%). Constitutively expressed *SAND1* and *YLS8* genes, which do not respond to changes in the Fe conditions (Han et al., 2013), were used as reference genes to normalize qRT-PCR results. The relative expression levels were calculated from the threshold cycles (Ct) values and the primer efficiencies by the Pfaffl method (Pfaffl, 2001). Each PCR analysis was conducted on three biological replicates and each PCR reaction repeated twice.

**Table 1:** Primer pairs for *Arabidopsis* genes.

**Gene**	**Sequence 5^**′**^-3^**′**^-**
*FRO2*	Forward: TGGTTGCCACATCTGCGTAT
(At1g01580)	Reverse: TCGATATGGTGTGGCGACTT
*IRT1*	Forward: TGTCTCTTTTGCAATCTGTCCA
(At4g19690)	Reverse: AGGAGCTCCAACACCAATCA
*FIT*	Forward: CCCTGTTTCATAGACGAGAACC
(At2g28160)	Reverse: TTCATCTTCTTCACCACCGGC
*PAP17*	Forward: CCTCCAAGTACGTTTCATCGATCC
(At3g17790)	Reverse: CCGTGGCGGACATTAACG
*PHT1;5*	Forward: TGTTGGCTCCACTGTTCCTC
(At2g32830)	Reverse: CGAGCAGTTTCCGGCATTTT
*PHR1*	Forward: TGCCAGGAGGGTTTCTTGAC
(At4g28610)	Reverse: GTCATCAAAAGCAGCCGCAA
*SULTR1;1*	Forward: GCCATCACAATCGCTCTCCAA
(At4g08620)	Reverse: TTGCCAATTCCACCCATGC
*SLIM1/EIL3*	Forward: GCCTTTGGGGGTTGGGTTAT
(At1g73730)	Reverse: ATGGCTGCGAATTGTGGAGA

### Statistical Analysis

All experiments were repeated at least twice and representative results are presented. The values of qRT-PCR represent the mean of three independent biological replicates. The values of FRA represent the mean of six replicates. Within each day and genotype, ^*^P < 0.05 or ^**^P < 0.01 indicate significant differences in relation to the control treatment using one-way analysis of variance (ANOVA) followed by a Dunnett's test.

## Results

In this work, different Fe, P, and S deficiency physiological responses have been examined along time in *Arabidopsis* WT Columbia and some of its ET signaling mutants (*ctr1, ein2-1*, and *ein3eil1*) subjected to the three deficiencies. The physiological responses studied include ferric reductase activity, phosphatase activity, and Fe-, P-, and S-related genes associated with the responses. Results corresponding to each particular deficiency are presented separately. It should be noted that plants did not show visible symptoms of deficiency in any of the treatments.

### Fe Deficiency Responses

**Figure 1:**
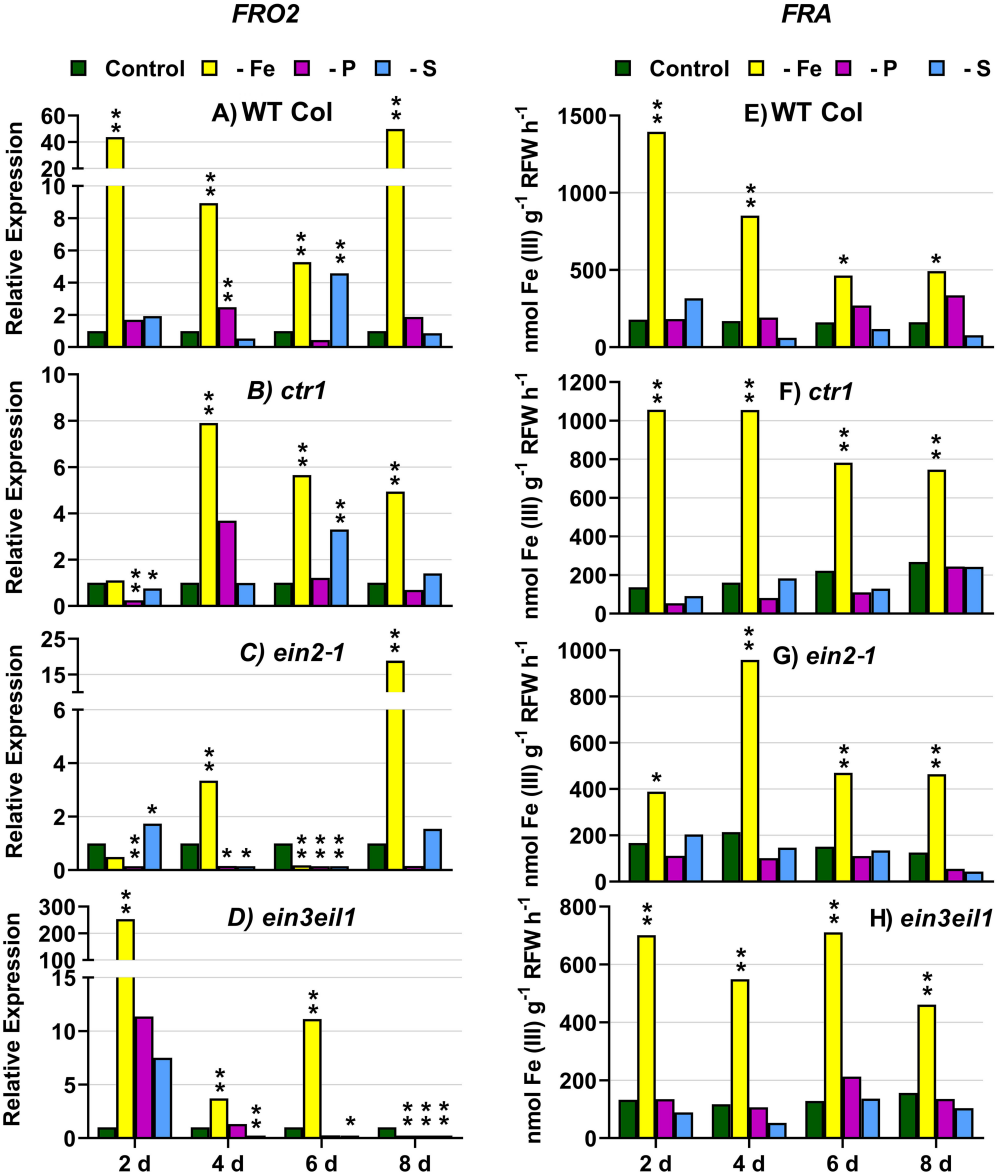
Effect of Fe, P, or S deficiency on the expression of the Fe-related gene *FRO2* (left: **A**–**D**) and of ferric reductase activity (FRA; right: **E**–**H**) in roots of the *Arabidopsis* WT Columbia and its ethylene mutants *ctr1, ein2-1*, and *ein3eil1*. Plants were grown in complete nutrient solution. When appropriate, some of them were transferred to complete nutrient solution (control), nutrient solution without Fe (–Fe), without P (–P), or without S (–S). After 2, 4, 6, and 8 days of the treatments, FRA and *FRO2* expression were determined. Relative expression was calculated in relation to the control of each genotype and day. Data of *FRO2* expression represent the mean of three independent biological replicates. Data of FRA represent the mean of six replicates. Within each day and genotype, *P < 0.05 or ^**^P < 0.01 are significant differences in relation to the control treatment.

**Figure 2:**
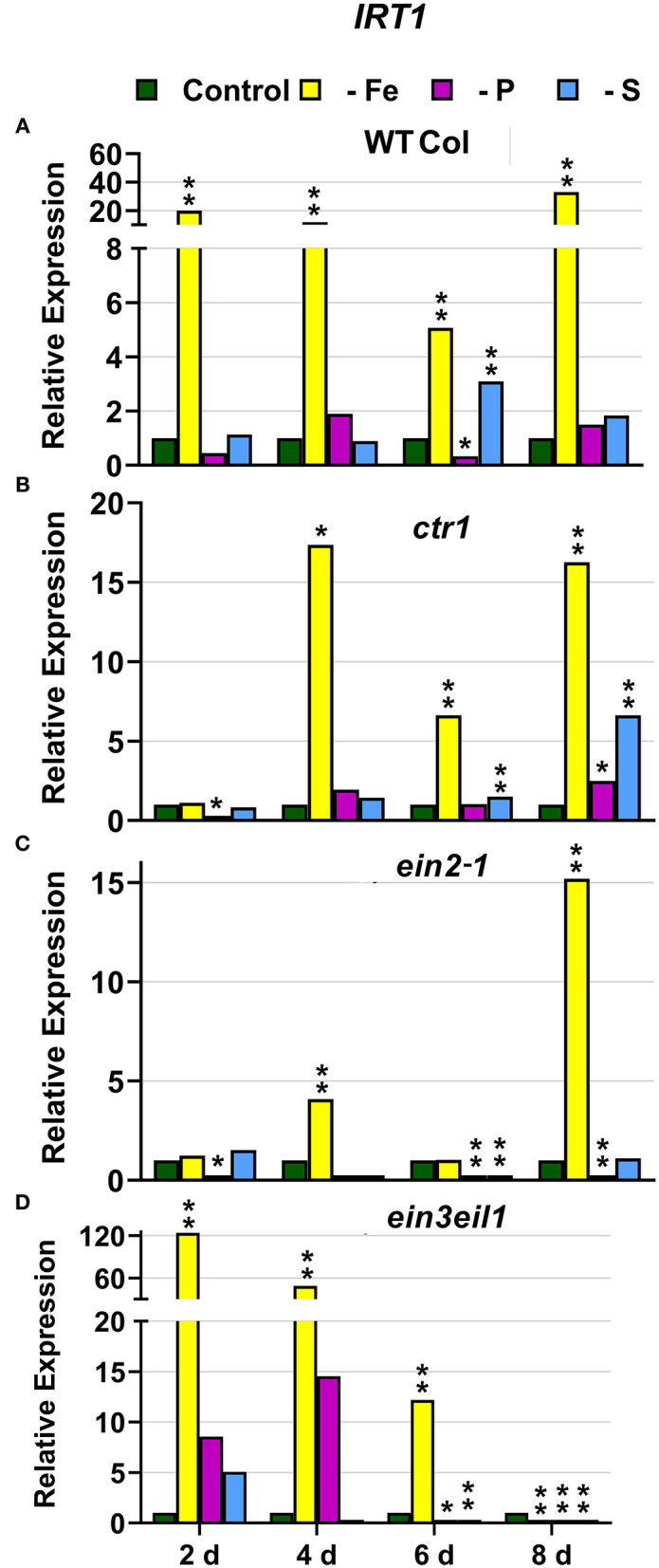
Effect of Fe, P or S deficiency on the expression of the Fe-related gene *IRT1* in roots of the *Arabidopsis* WT Columbia and its ethylene mutants *ctr1, ein2-1* and *ein3eil1* **(A–D)**. Treatments and gene expression determination as in Figure 1. Within each day and genotype, * or ** indicate significant differences (*P* < 0.05 or *P* < 0.01) in relation to the control treatment.

**Figure 3:**
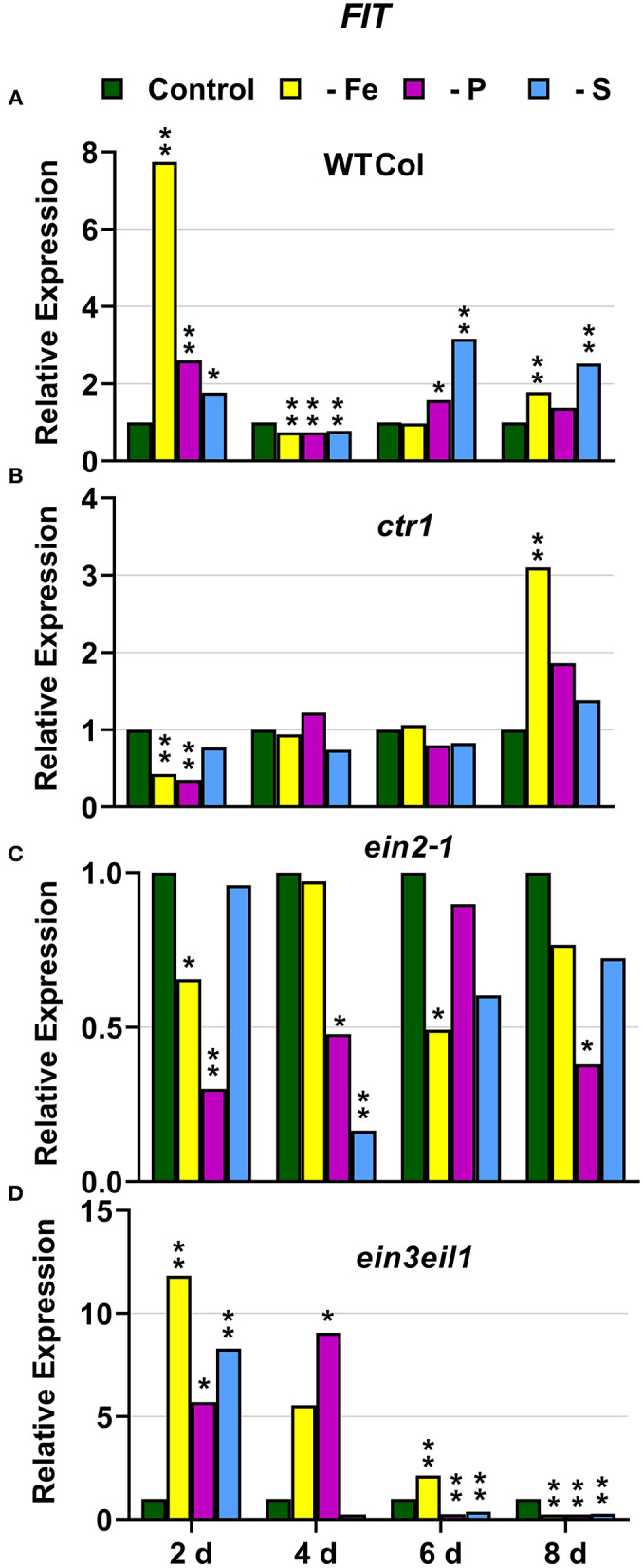
Effect of Fe, P or S deficiency on the expression of the Fe-related gene *FIT* in roots of the *Arabidopsis* WT Columbia and its ethylene mutants *ctr1, ein2-1* and *ein3eil1* **(A–D)**. Treatments and gene expression determination as in Figure 1. Within each day and genotype, * or ** indicate significant differences (*P* < 0.05 or *P* < 0.01) in relation to the control treatment.

In WT Columbia plants, as expected, *FRO2, IRT1*, and *FIT* expression, and ferric reductase activity (FRA; associated with *FRO2*), were greatly induced after 2 days of Fe deficiency. Later on, the induction decayed and tended to recover some days after the deficiency (Figures 1–3). In relation to the other deficiencies, most of the Fe-related genes (*FRO2, IRT1*, and *FIT*) were also induced in WT Columbia plants under both P and S deficiency but to a much lower extent than under the Fe deficiency itself (Figures 1–3). However, FRA was not appreciably induced in WT Columbia plants under either P or S deficiency (Figure 1E).

In the *ctr1* mutant, FRA was also greatly induced after 2 days of Fe deficiency while the Fe-related genes (*FRO2, IRT1*, and *FIT*) were also induced but after 4 days of the Fe deficiency and, in general, to lower levels than in the WT Columbia (Figures 1–3). In relation to the other deficiencies, neither FRA nor *FIT* were induced under either P or S deficiency while *FRO2* and *IRT1* were slightly induced under S deficiency (Figures 1–3).

In the *ein2-1* mutant, *FRO2*, FRA, and *IRT1* induction under Fe deficiency were delayed in relation to the WT Columbia and, in general, the maximum values achieved were lower than in the WT (Figures 1, 2). Surprisingly, despite *FRO2* and *IRT1* induction under Fe deficiency, *FIT* expression was not upregulated in this mutant at any time studied (Figures 1–3). In relation to the other deficiencies, FRA was not induced in this mutant under either P or S deficiency (Figure 1G). Similarly to the results obtained under Fe deficiency, neither *FIT* nor the Fe acquisition genes *FRO2* and *IRT1* were clearly upregulated in the *ein2-1* mutant under either P or S deficiency (only *FRO2* was slightly induced after 2 days of S deficiency; Figures 1–3).

In the *ein3eil1* mutant, as occurred in the WT Columbia, *FRO2*, FRA, *IRT1*, and *FIT* were greatly induced after 2 days of Fe deficiency. It should be noted that the maximum values of *FRO2, IRT1*, and *FIT* expression were attained in this mutant, where some values were several times those obtained in the WT Columbia (Figures 1–3). In relation to the other deficiencies, neither FRA nor the Fe acquisition genes *FRO2* and *IRT1* were significantly induced under either P or S deficiency (Figures 1, 2). However, *FIT* was upregulated under both P and S deficiency in this mutant (Figure 3D).

Collectively, the results show that Fe deficiency responses can also be induced under P or S deficiency but to lower intensities and more transiently. The induction of the Fe deficiency responses under Fe deficiency itself or under P or S deficiency differs depending on the ET signaling mutants. However, FRA was only induced by Fe deficiency itself but neither by P deficiency nor by S deficiency in any of the genotypes studied.

### P Deficiency Responses

**Figure 4:**
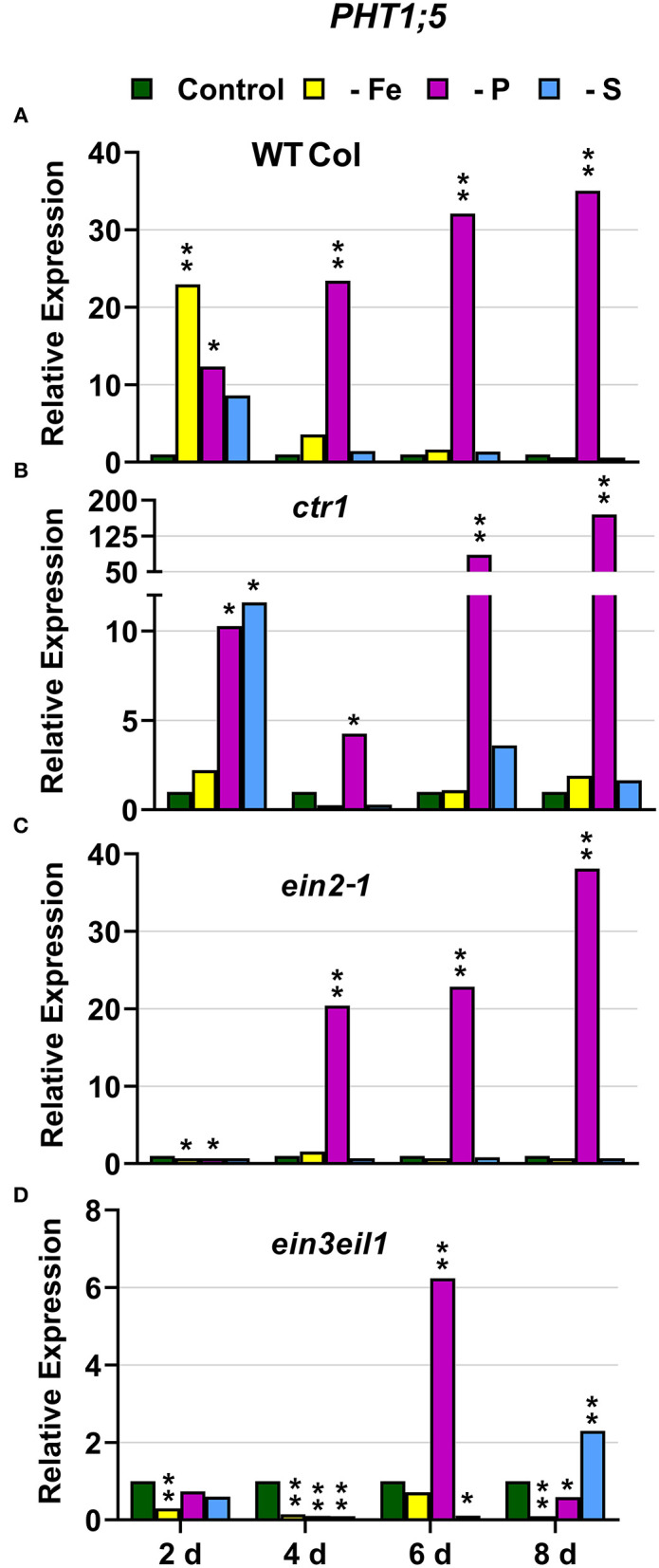
Effect of Fe, P or S deficiency on the expression of the P-related gene *PHT1;5* in roots of the *Arabidopsis* WT Columbia and its ethylene mutants *ctr1, ein2-1* and *ein3eil1* **(A–D)**. Treatments and gene expression determination as in Figure 1. Within each day and genotype, * or ** indicate significant differences (*P* < 0.05 or *P* < 0.01) in relation to the control treatment.

**Figure 5:**
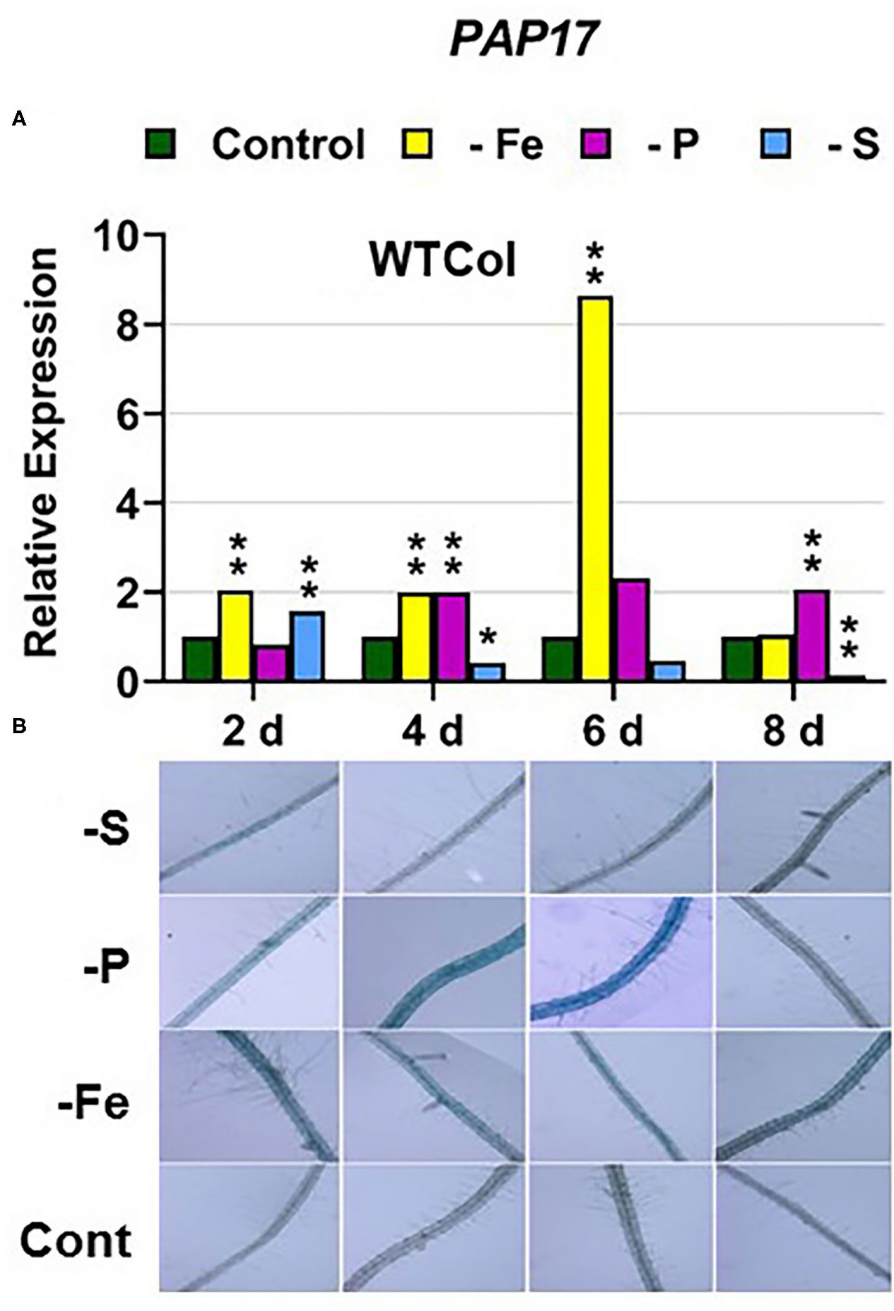
Effect of Fe, P or S deficiency on the expression of the P-related gene *PAP17* **(A)**; and of phosphatase activity (PA; **B**) in roots of the *Arabidopsis* WT Columbia. Treatments and gene expression determination as in Figure 1. After 2, 4, 6, and 8 days of the treatments, PA was determined in several plant replications and representative results are presented. Within each day and genotype, * or ** indicate significant differences (*P* < 0.05 or *P* < 0.01) in relation to the control treatment.

In WT Columbia plants, *PHT1;5, PAP17*, and *PHR1* expression, and phosphatase activity (PA; associated with *PAP* genes), were induced after 2–4 days of P deficiency (Figures 4A, 5, **9A**). In relation to the other deficiencies, *PHT1;5* was also induced under Fe deficiency (Figure 4A). *PAP17* was also induced under both Fe and S deficiency while PA was induced by Fe deficiency but not by S deficiency (Figure 5). *PHR1* was also upregulated by both Fe and S deficiency (**Figure 9A**).

**Figure 6:**
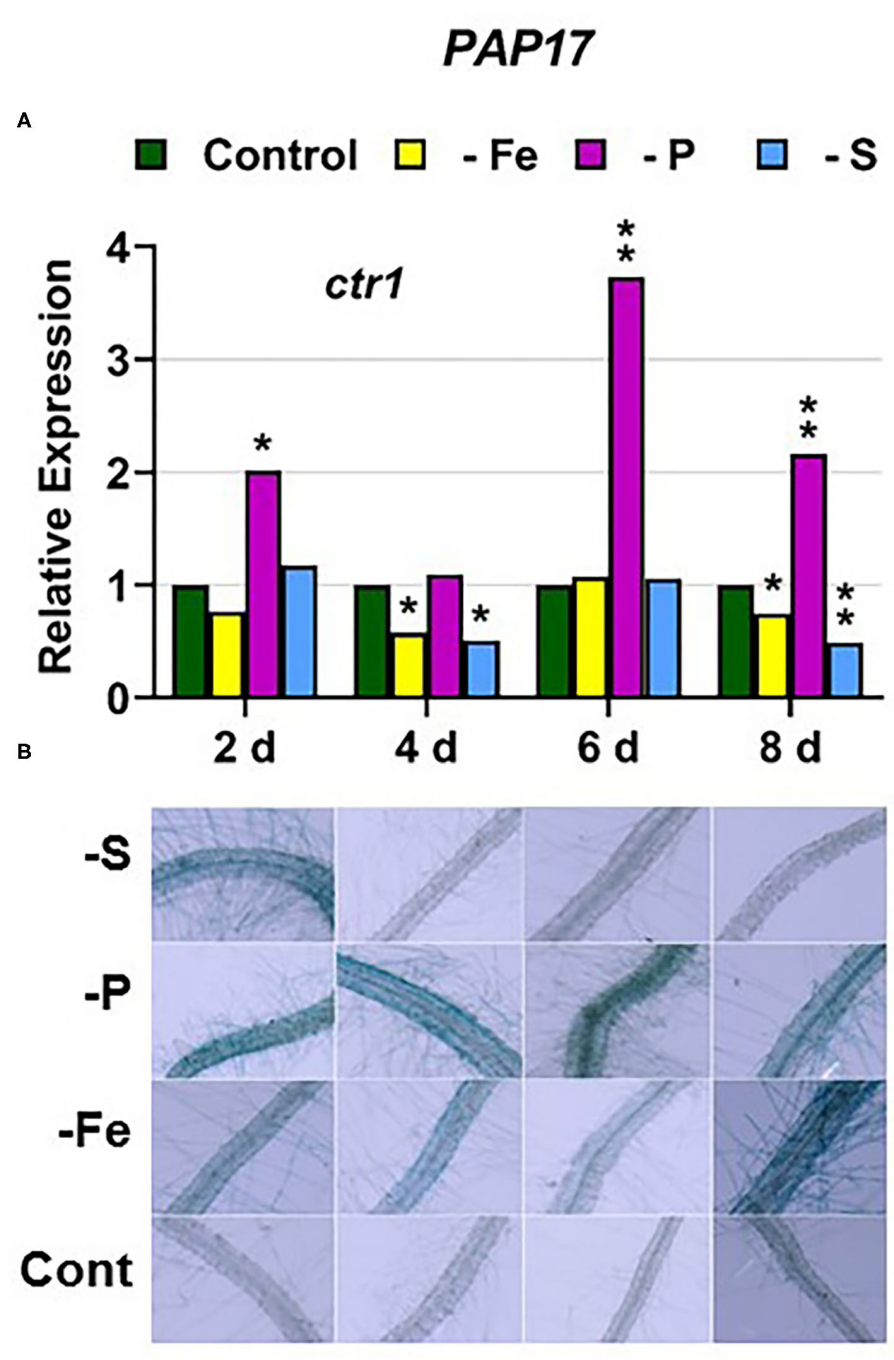
Effect of Fe, P or S deficiency on the expression of the P-related gene *PAP17* **(A)**; and of phosphatase activity (PA; **B**) in roots of the *Arabidopsis* ethylene mutant *ctr1*. Treatments and determinations as in Figure 5. Within each day and genotype, * or ** indicate significant differences (*P* < 0.05 or *P* < 0.01) in relation to the control treatment.

In the *ctr1* mutant, *PHT1;5* expression under P deficiency achieved the highest values of its induction (Figure 4). PA, *PAP17*, and *PHR1* were also induced in this mutant under P deficiency (Figures 6, **9**). In relation to the other deficiencies, *PHT1;5* was also induced under S deficiency (Figure 4B). PA was also induced under both Fe and S deficiencies while *PAP17* was not (Figure 6). *PHR1* was not induced by Fe deficiency, but it was slightly upregulated by S deficiency (**Figure 9B**).

**Figure 7:**
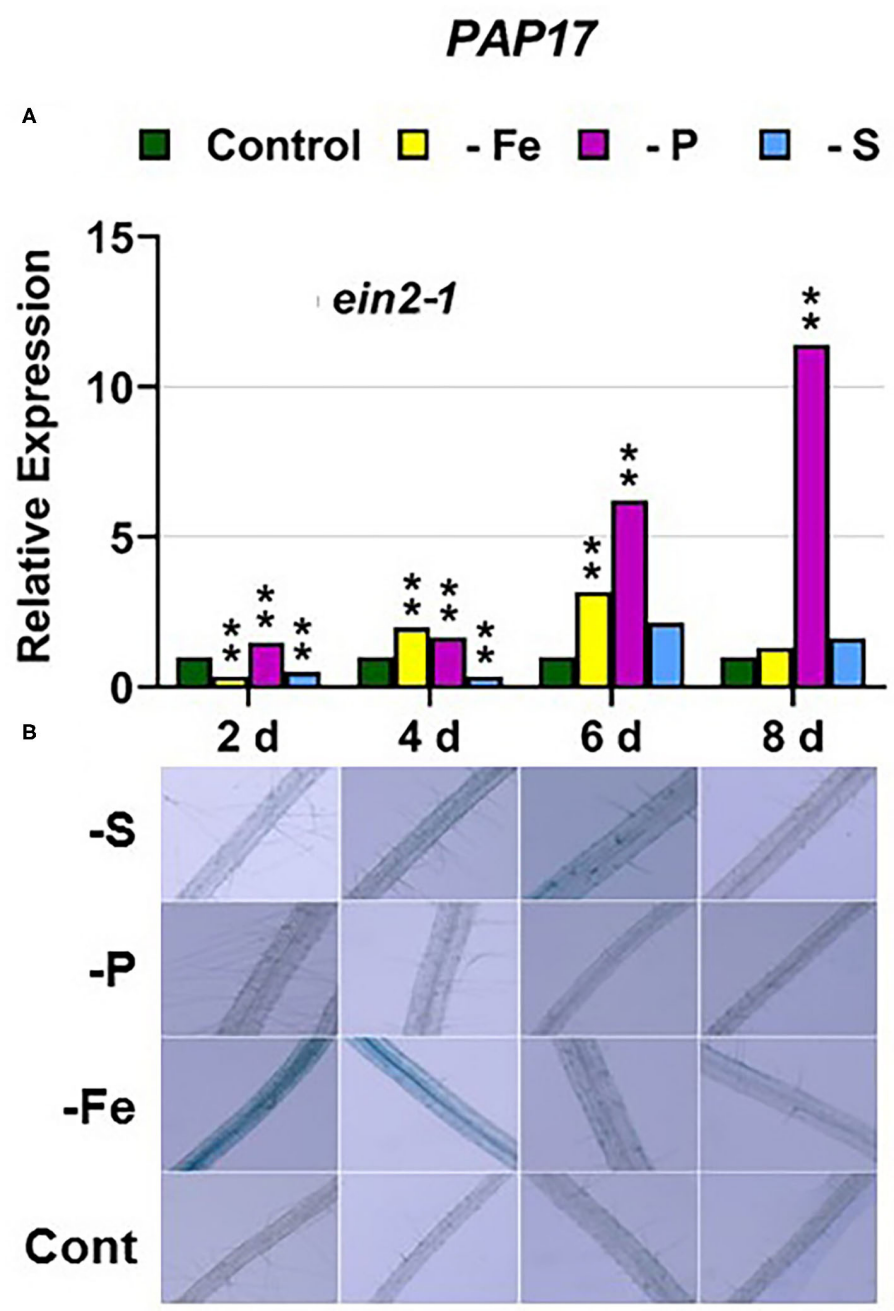
Effect of Fe, P or S deficiency on the expression of the P-related gene *PAP17* **(A)**; and of phosphatase activity (PA; **B**) in roots of the *Arabidopsis* ethylene mutant *ein2-1*. Treatments and determinations as in Figure 5. Within each day and genotype, * or ** indicate significant differences (*P* < 0.05 or *P* < 0.01) in relation to the control treatment.

In the *ein2-1* mutant, *PHT1;5* was induced under P deficiency (Figure 4C). In this mutant, *PAP17* expression was induced under P deficiency but PA was not (Figure 7). *PHR1* was also upregulated under P deficiency but after 6 days of P deficiency and to lower levels than in the other genotypes (**Figure 9**). In relation to the other deficiencies, neither *PHT1;5* nor *PHR1* were induced under either Fe or S deficiency in the *ein2-1* mutant (Figures 4C, **9C**). However, both PA and *PAP17* were induced under Fe deficiency in the *ein2-1* mutant (Figure 7).

**Figure 8:**
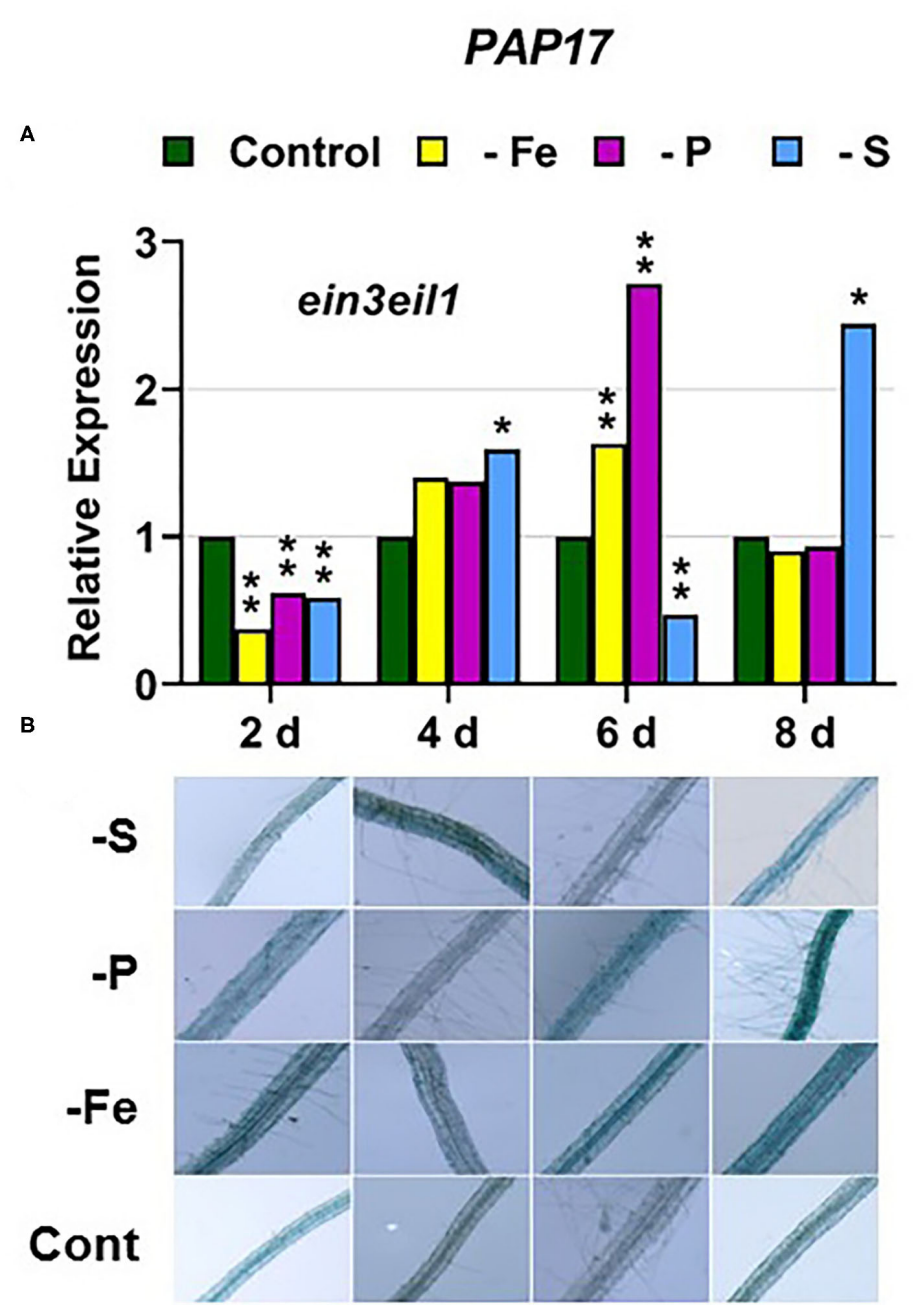
Effect of Fe, P or S deficiency on the expression of the P-related gene *PAP17* **(A)**; and of phosphatase activity (PA; **B**) in roots of the *Arabidopsis* ethylene mutant *ein3eil1*. Treatments and determinations as in Figure 5. Within each day and genotype, * or ** indicate significant differences (*P* < 0.05 or *P* < 0.01) in relation to the control treatment.

**Figure 9:**
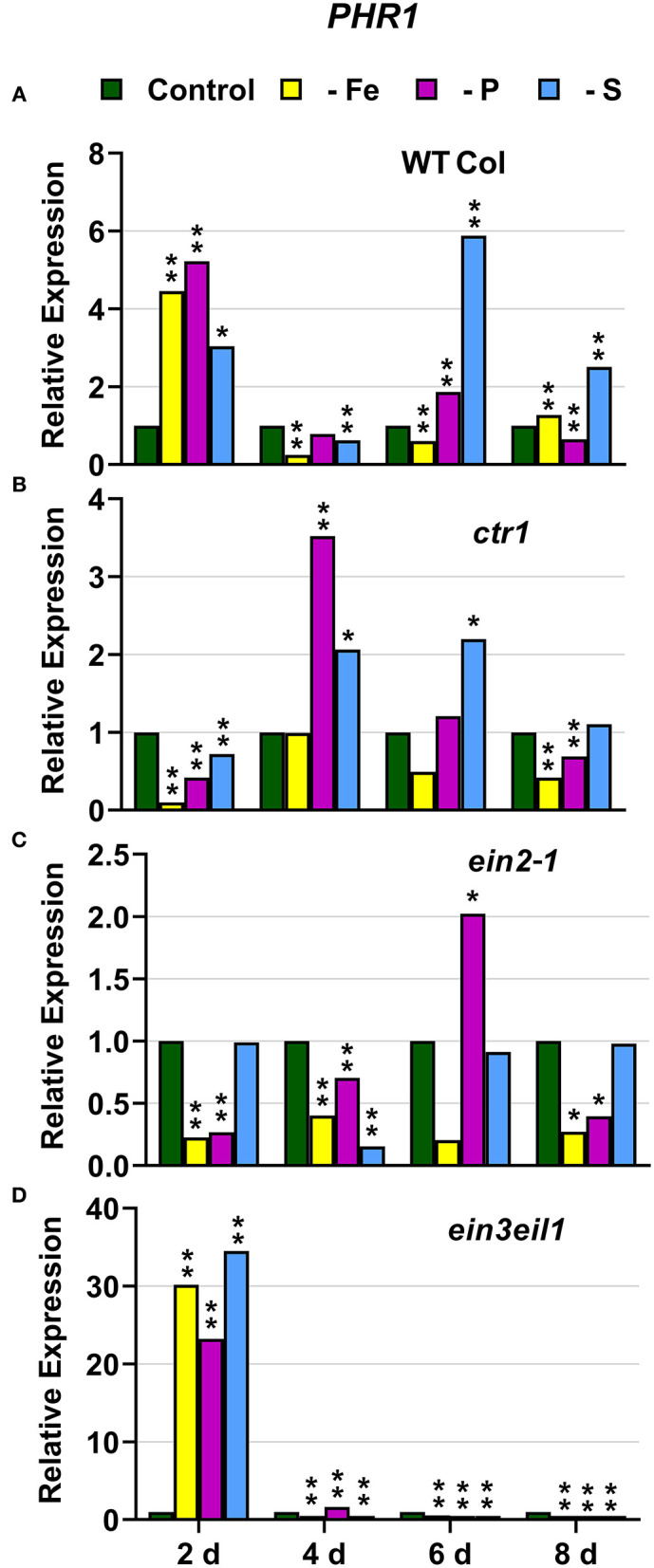
Effect of Fe, P or S deficiency on the expression of the P-related gene *PHR1* in roots of the *Arabidopsis* WT Columbia and its ethylene mutants *ctr1, ein2-1* and *ein3eil1* **(A–D)**. Treatments and gene expression determination as in Figure 1. Within each day and genotype, * or ** indicate significant differences (*P* < 0.05 or *P* < 0.01) in relation to the control treatment.

In the *ein3eil1* mutant, *PHT1;5* expression was delayed, starting after 6 days of the P deficiency, attaining the lowest values within the different genotypes, while *PHR1* expression reached its highest level of induction (Figures 4, **9**). Both PA and *PAP17* were also induced under P deficiency (Figure 8). In relation to the other deficiencies, *PHT1;5* was induced after 8 days of S deficiency (Figure 4D). PA, *PAP17*, and *PHR1* were induced under both Fe and S deficiencies (Figures 8, 9D).

Collectively, the results show that P deficiency responses can also be induced under Fe or S deficiency. In this case, some P deficiency responses attained similar or higher intensities under Fe deficiency, and in some cases under S deficiency, than under P deficiency itself: see, for example, *PAP17* expression and PA under Fe deficiency (Figures 5, 8). The induction of the P deficiency responses under P deficiency itself or under Fe or S deficiency differs depending on the ET signaling mutants.

### S Deficiency Responses

**Figure 10:**
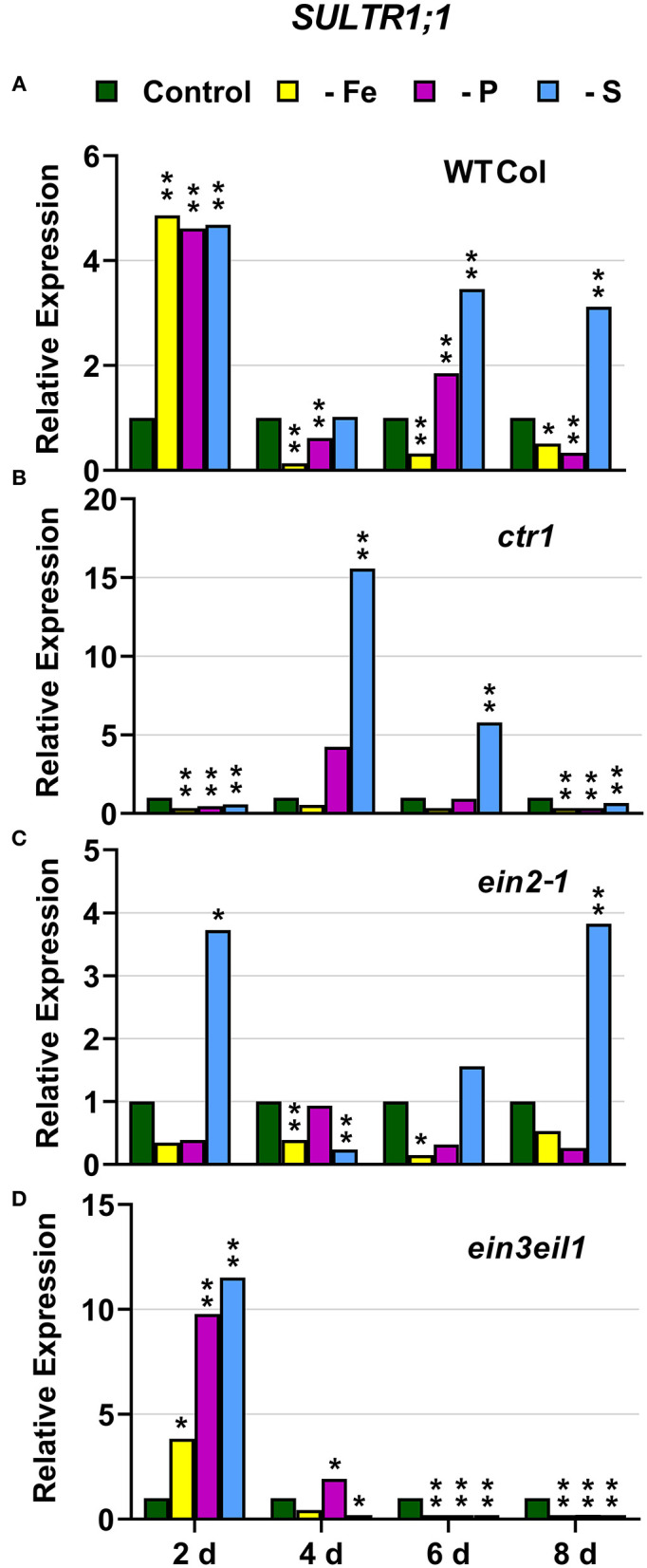
Effect of Fe, P or S deficiency on the expression of the S-related gene *SULTR1;1* in roots of the *Arabidopsis* WT Columbia and its ethylene mutants *ctr1, ein2-1* and *ein3eil1* **(A–D)**. Treatments and gene expression determination as in Figure 1. Within each day and genotype, * or ** indicate significant differences (*P* < 0.05 or *P* < 0.01) in relation to the control treatment.

**Figure 11:**
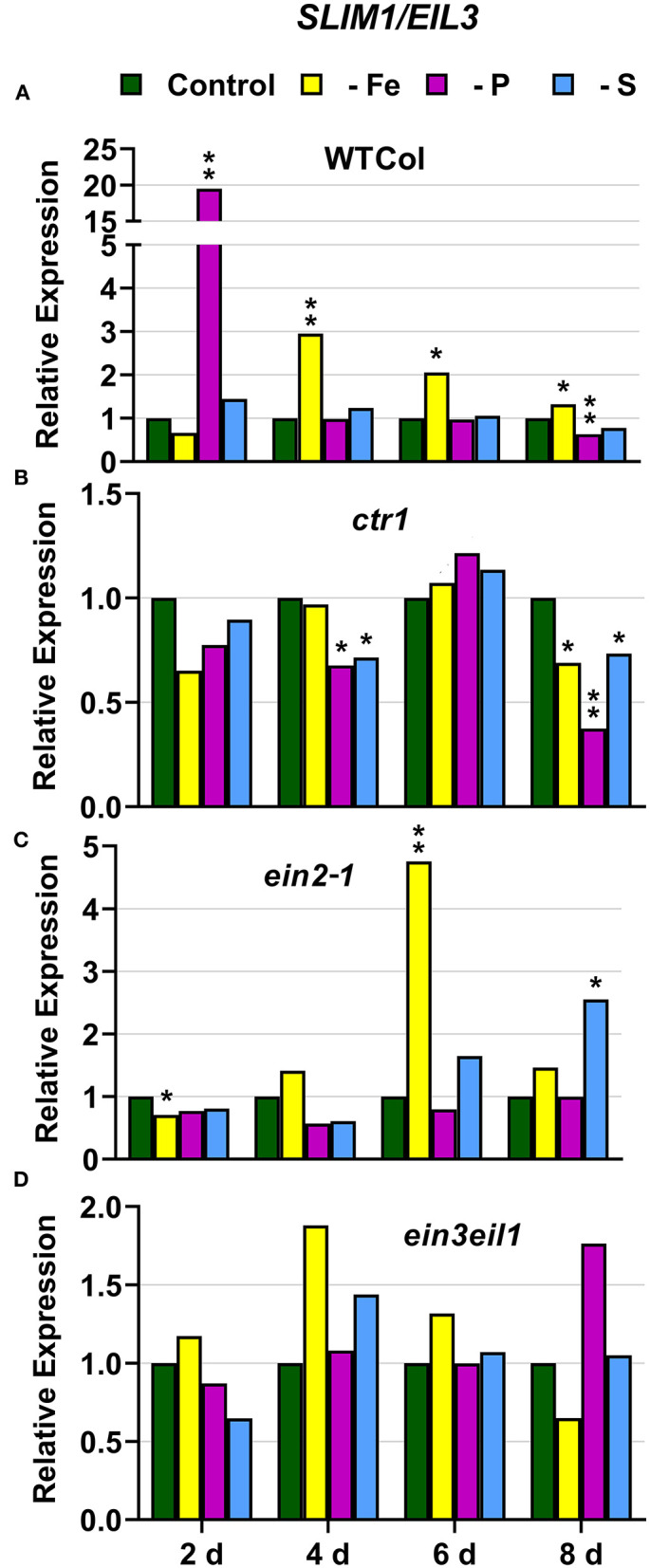
Effect of Fe, P or S deficiency on the expression of the S-related gene *SLIM1/EIL3* in roots of the *Arabidopsis* WT Columbia and its ethylene mutants *ctr1, ein2-1* and *ein3eil1* **(A–D)**. Treatments and gene expression determination as in Figure 1. Within each day and genotype, * or ** indicate significant differences (*P* < 0.05 or *P* < 0.01) in relation to the control treatment.

In WT Columbia plants, *SULTR1;1* expression was upregulated after 2 days of S deficiency. By contrast, *SLIM1/EIL3* was not upregulated under S deficiency at any time. In relation to the other deficiencies, both *SULTR1;1* and *SLIM1/EIL3* were upregulated under both P and Fe deficiency (Figures 10A, 11A).

In the *ctr1* mutant, *SULTR1;1* reached the highest values of its expression under S deficiency while *SLIM1/EIL3* was not upregulated by S deficiency. In relation to the other deficiencies, neither *SULTR1;1* nor *SLIM1/EIL3* were upregulated by either Fe or P deficiency (Figures 10B, 11B)

In the *ein2-1* mutant, *SULTR1;1* was upregulated after 2 days of S deficiency while *SLIM1/EIL3* was slightly upregulated but only after 8 days of the deficiency. In relation to the other deficiencies, *SULTR1;1* was not upregulated by either Fe or P deficiency while *SLIM1/EIL3* was upregulated under Fe deficiency (Figures 10C, 11C).

In the *ein3eil1* mutant, *SULTR1;1* was upregulated after 2 days of S deficiency while *SLIM1/EIL3* was not upregulated under S deficiency at any time. In relation to the other deficiencies, *SULTR1;1* was upregulated by both Fe and P deficiency while *SLIM1/EIL3* was not (Figures 10D, 11D).

Collectively, the results show that *SULTR1;1* expression (a S deficiency response) can also be upregulated under Fe or P deficiency in the WT Columbia and in the *ein3eil1* mutant (Figure 10). In the case of *SLIM1/EIL3*, the results obtained in this work show its lack of induction upon S deficiency in the WT Columbia. However, it can be upregulated under S, Fe, or P deficiency depending on the ET-related genotype of the plants (Figure 11).

## Discussion

The Fe-related genes (*FIT, FRO2*, and *IRT1*), the P-related genes (*PHR1, PHT1;5*, and *PAP17*) and the S-related gene *SULTR1;1* were induced by their respective deficiencies in the WT Columbia (Figures 1–10), which agrees with already published results (see “Introduction”). Only the S-related gene *SLIM1/EIL3* was not induced by its corresponding deficiency (Figure 11), which also coincides with previous results (Maruyama-Nakashita et al., 2006). In the case of *PHR1*, although its upregulation under P deficiency is controversial (Rubio et al., 2001; Sega and Pacak, 2019), our results show it is upregulated (Figure 9), as in Huang et al. (2018). In addition to the WT Columbia, most of the above genes were also induced under their respective deficiencies in the ET signaling mutants used but with different intensities and at different times than in the WT (Figures 1–11). It should be noted the importance of timing in the results (Harkey et al., 2018) since the maximum expression of the genes was frequently reached at different times depending on the genotypes (Figures 1–11).

Besides the upregulation of the genes by their specific deficiencies, most of the genes were also upregulated by the other deficiencies, which confirms previous published results (see “Introduction”). In any case, the upregulation was differentially affected by the ET signaling mutations, as discussed below. This further supports a key role for ET in the regulation of Fe, P, and S deficiency responses and in the crosstalk between them. To our knowledge, it is the first time that ET and some key components of its signaling pathway, such as EIN2 and EIN3/EIL1, are involved in such crosstalk.

In most cases, the highest values of induction in the WT Columbia plants were reached by the specific deficiency: for example, the highest values of *FRO2, IRT1*, and *FIT* expression were obtained under Fe deficiency and those of *PHT1;5* expression under P deficiency (Figures 1–4). There are two clear exceptions to this trend: the highest values of *PAP17* expression under Fe deficiency and those of *SLIM1/EIL3* expression under P and Fe deficiency (Figures 5A, 11A). Another difference between the induction of the responses by specific and nonspecific deficiencies is that the induction provoked by a nonspecific deficiency is more transitory. For example, *PHT1;5* and *SULTR1;1* expression in WT Columbia plants under Fe deficiency was intense after 2 days of the deficiency but then decayed drastically for the remainder of the experiment (Figures 4A, 10A). These results suggest that, for the regulation of the responses, ET should act in conjunction with nutrient-specific repressive signals (Lucena et al., 2006, 2015; Lei et al., 2011; García et al., 2013, 2015, 2018; Romera et al., 2017). In the presence of such nutrient-specific repressive signals, ET could help to induce the responses but they would be quickly repressed to avoid the excessive uptake of the nutrient that is already in adequate concentrations inside the plant.

In relation to FRA and PA activities, their behavior was completely different. While FRA was induced in the WT Columbia and in all the mutants only under Fe deficiency (Figure 1), PA was also induced by both Fe and S deficiencies, besides P deficiency, depending on the genotypes (Figures 5B–8B). The lack of induction of FRA under P or S deficiency, despite *FRO2* upregulation under both deficiencies (Figure 1), suggests the existence of a post-transcriptional regulation of *FRO2* depending on Fe-related repressive signals (Connolly et al., 2003). The possible post-transcriptional regulation of the *PAP* genes seems to be less dependent on the existence of P-related repressive signals since PA is clearly induced under the other deficiencies, mainly under Fe deficiency (Figures 5–8). The tight post-transcriptional regulation of *FRO2* is probably associated with the fact that Fe^2+^ in excess is highly reactive and potentially toxic (Mendoza-Cózatl et al., 2019). However, P in excess is less toxic and consequently the post-transcriptional regulation of *PAP* genes is not so critical.

Besides the participation of nutrient-specific repressive signals, another possibility to explain the differential role of ET in the regulation of responses to the three deficiencies is to consider its action through different signaling pathways. In fact, nutrient deficiencies can affect ET responsiveness by altering the expression of genes involved in ET signaling, such as *CTR1, EIN2, EIN3*, and *ERFs* (García et al., 2010, 2015; Lucena et al., 2015; Song and Liu, 2015; Li and Lan, 2017; Romera et al., 2017). To test the involvement of ET signaling in the regulation of Fe, P, and S deficiency responses, and in the crosstalk between them, we have studied their induction in the *Arabidopsis* WT Columbia and in three of its ET signaling mutants: the *ctr1* mutant, that presents constitutive ET responses; and the *ein2-1* and *ein3eil1* mutants, that are described as insensitive to ET (Alonso et al., 1999; Guo and Ecker, 2003; Huang et al., 2003; Shakeel et al., 2013; Wang et al., 2013; Dubois et al., 2018; Binder, 2020). In the following paragraphs, the results obtained in this work in relation to the CTR1, EIN2, and EIN3/EIL1 components of the ET signaling pathway are discussed separately.

### CTR1

The *ctr1* mutant displays the known “triple-response” morphology in the absence of exogenously added ET (Huang et al., 2003). In relation to CTR1, the results presented in this work (Figures 1–4, 6, 9–11) show that the *ctr1* mutant does not present constitutive upregulation of any of the Fe-, P-, and S-related genes studied, all of them are upregulated upon the imposition of the deficiencies. In the same way, this mutant does not show constitutive activation of either FRA or PA (Figures 1F, 6B). These results agree with previous ones showing that *PHT1;4* (*PT2*) is not constitutively upregulated in the *hsp2* (*ctr1*) mutant (Lei et al., 2011) and that Fe-related genes, like *FRO2, IRT1*, and *FIT*, and FRA, are not constitutively induced in the *ctr1* mutant (García et al., 2014). Taken together, all these results suggest that, for the activation of physiological responses by ET, a defective CTR1 is not enough and an internal decrease of some nutrient-specific repressive signals would be necessary. This decrease would not be required for some morphological responses, like the development of subapical root hairs, since the *ctr1* mutant does present them even when grown in complete nutrient solution (Romera and Alcántara, 2004).

In relation to the crosstalk between the three deficiencies, CTR1 seems to play a role since Fe deficiency did not induce either the P-related genes *PHT1;5, PAP17*, and *PHR1* or the S-related genes *SULTR1;1* and *SLIM1/EIL3* in the *ctr1* mutant while it did in the WT Columbia (Figures 4–6, 9–11). The reasons for this are not clear and would need further research. In addition, PA was induced by Fe deficiency in the *ctr1* mutant despite *PAP17* was not (Figure 6). This result could be explained by the induction of other *PAP* genes, besides *PAP17*, encoding phosphatases (Sun et al., 2016).

### EIN2

**Figure 12:**
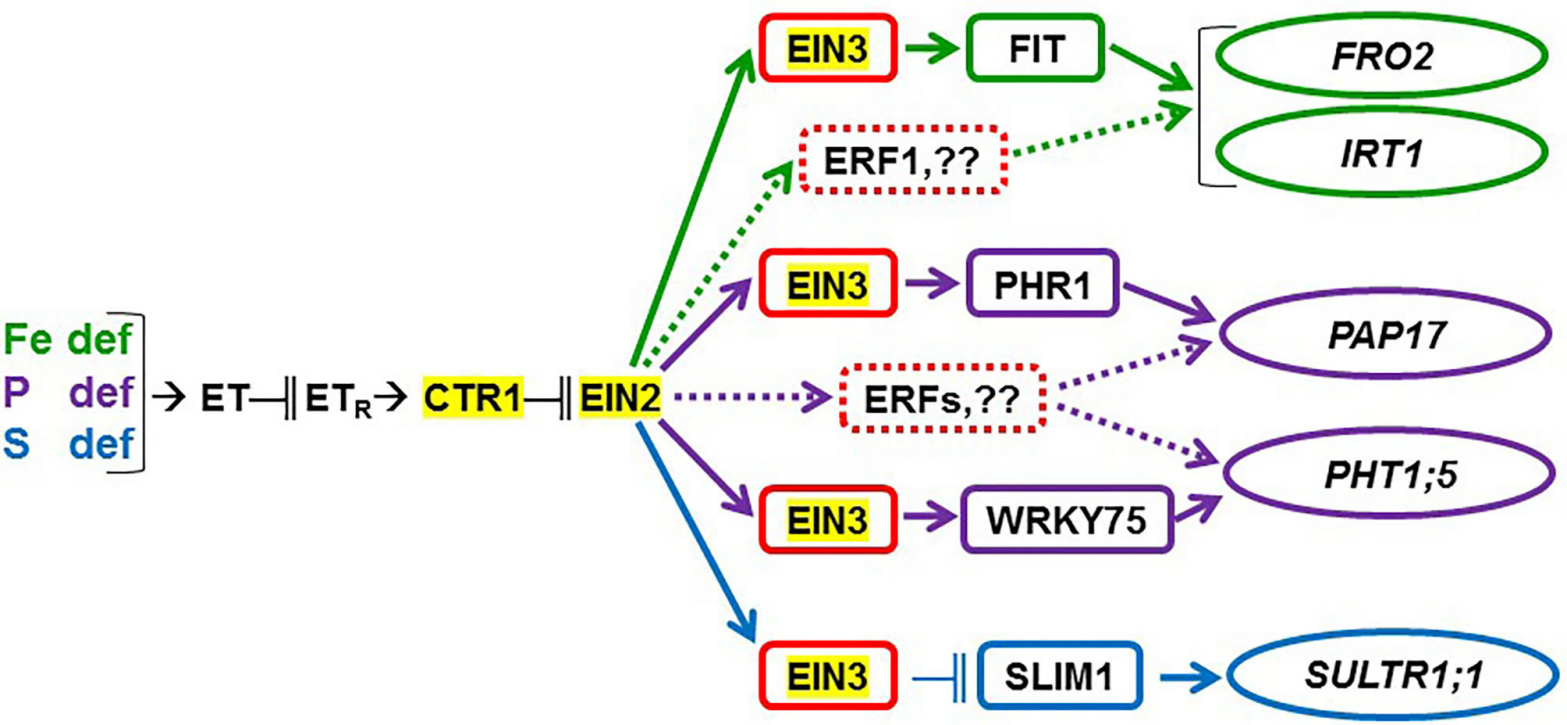
Possible participation of ethylene, through CTR1, EIN2, and EIN3/EIL1, in the regulation of the Fe-, P-, and S-related genes considered in this study. Ethylene (ET) production is enhanced in Fe-, P-, and S-deficient roots. ET then acts in a signaling pathway which includes ET receptors (ET_R_) and the CTR1 and EIN2 proteins. This latter one can act through the EIN3/EIL1 TFs (represented as EIN3 in the figure), which have been implicated in the regulation of the FIT, PHR1, WRKY75, and SLIM1 TFs controlling the activation of Fe-, P-, and S-related genes, like *FRO2, IRT1, PAP17, PHT1;5*, and *SULTR1;1* (see text for details). Results in this work suggest that the EIN2 protein plays a key role in the regulation of the physiological responses to the three deficiencies and in the crosstalk between them. Moreover, the results support that TFs in addition to EIN3/EIL1, like ERF1 and other ERFs, could participate in the upregulation of Fe- and P-related genes (dotted lines). In red, ET-related TFs; in green, Fe-related TF and genes; in purple, P-related TFs and genes; in blue, S-related TF and gene. “ → ” promotion; “−∥” inhibition.

The *ein2-1* mutant has been described as insensitive in most responses to ET (Alonso et al., 1999; Shakeel et al., 2013; Wang et al., 2013; Dubois et al., 2018; Binder, 2020). In fact, EIN2 has been considered one of the central players in the linear canonical signaling pathway proposed for ET action (see Introduction; Wang et al., 2013; Dubois et al., 2018; Binder, 2020). In relation to each particular deficiency, *FIT* (encoding a key TF) was not induced under Fe deficiency (Figure 3C) while *PHR1* (also encoding a key TF) was only slightly induced under P deficiency in the *ein2-1* mutant (Figure 9C). Despite these results, the genes activated by both TFs, such as *FRO2* and *IRT1*, and *PAP17*, were upregulated under Fe deficiency or P deficiency, respectively (Figures 1C, 2C, 7A). In spite of *PAP17* induction, PA was not induced under P deficiency in this mutant (Figure 7B), which conforms to previous results showing the impairment of PA induction in the *ein2-5* mutant under P deficiency (Lei et al., 2011). All these results could be partly explained by taking into account that both *FIT* and *PHR1*, and perhaps *PAP17*, can also be subjected to post-transcriptional and post-translational modifications (Lingam et al., 2011; Jung et al., 2018; Sega and Pacak, 2019; Wu and Ling, 2019). Another possibility could be the existence of additional FIT-and PHR1-independent pathways to control the expression of genes like *FRO2, IRT1*, and *PAP17* (Figure 12).

In relation to the crosstalk between the three deficiencies, EIN2 probably plays a key role on it because the Fe-related gene *IRT1*, the P-related gene *PHT1;5*, and the S-related gene *SULTR1;1* were not appreciably induced in the *ein2-1* mutant under nonspecific deficiencies (Figures 2, 4, 10). In the case of Fe and P deficiencies, the relevant role of EIN2 in the crosstalk between them is further supported by the results showing its great influence on the upregulation of the genes encoding the key TFs *FIT* (Fe) and *PHR1* (P). Neither *FIT* was induced under P deficiency nor *PHR1* was induced under Fe deficiency in the *ein2-1* mutant, as occurred in the WT Columbia (Figures 3, 9).

Besides its role in the crosstalk between Fe and P deficiencies through its effects on *FIT* and *PHR1* expression (see above), EIN2 could also affect other genes not activated by these TFs, like *PHT1;5*, encoding an internal P transporter (Nagarajan et al., 2011). *PHT1;5* is not activated by the PHR1 TF but by the WRKY75 TF (Nagarajan et al., 2011), which is also regulated by ET through EIN3/EIL1 (Figure 12; Guo et al., 2017). It seems that EIN2 plays an important role in the *PHT1;5* upregulation under nonspecific deficiencies, since *PHT1;5* was only induced under P deficiency in the *ein2-1* mutant while in the WT Columbia was also induced under Fe deficiency (Figure 4).

### EIN3/EIL1

Similar to the *ein2-1* mutant, the *ein3eil1* double mutant has also been considered insensitive in most responses to ET (Shakeel et al., 2013; Wang et al., 2013; Dubois et al., 2018; Binder, 2020), although it does not present complete insensitivity (Harkey et al., 2018). The results obtained in this work show that most genes considered in this study (except *SLIM1*) were upregulated in the *ein3eil1* double mutant under their specific deficiencies, as occurred in the WT Columbia (Figures 1–5, 8–11). These results coincide with previous ones showing upregulation of *FIT* under Fe deficiency (Lingam et al., 2011), and of *PAP17* (*ACP5*) under P deficiency (Liu et al., 2017), in the *ein3eil1* mutant. Moreover, several genes, like *FRO2, IRT1, FIT, PHR1*, and *SULTR1;1* attained their highest expression, and also the lowest one, in the *ein3eil1* mutant (Figures 1–3, 9, 10). PA was also greatly induced in this mutant under P deficiency (Figure 8B). In the case of *FIT* and *PHR1*, their highest expression was somewhat surprising since EIN3/EIL1 have been involved in the activation of *FIT* (Yang et al., 2014) and *PHR1* (Liu et al., 2017). A possible explanation for the above results could be the existence of additional EIN3/EIL1-independent pathways for the control of *FIT* and *PHR1* expression, and/or for the Fe and P acquisition genes controlled by them (Figure 12). The idea of an EIN3/FIT-independent pathway has already been proposed by Balparda et al. (2020), showing that *FRO2* and *IRT1* expression, besides its control by FIT, could also be directly controlled by the ERF1 TF (also associated with ET; see “Introduction”). The existence of EIN3/EIL1-independent pathways for the control of *PHR1*, and consequently for P acquisition genes, is also possible because several transcriptomic analyses have shown altered expression of *ERF* genes, like *ERF1, ERF2*, and *ERF5*, in Pi-starved *Arabidopsis* roots (Song and Liu, 2015 and references therein). The possibility exists that the EIN3/EIL1-independent pathways could be potentiated when the EIN3/EIL1-dependent pathway is impaired. This would explain why some nutrient-deficiency responses are greatly induced in the *ein3eil1* mutant (see above).

In relation to the crosstalk between the three deficiencies, the EIN3/EIL1 mutation, by contrast to the EIN2 mutation, did not impair the upregulation of most of the genes under nonspecific deficiencies (Figures 1–4, 8–11). This again suggests the existence of EIN3/EIL1-independent pathways for the control of Fe- and P-related genes (Figure 12). In the case of *SULTR1;1*, its higher upregulation under S deficiency, and also under P deficiency, in the *ein3eil1* mutant (Figure 10) could be partly explained by considering that EIN3 can negatively interact with SLIM1/EIL3 for the upregulation of S acquisition genes (Wawrzynska and Sirko, 2016). The results agree with those of Wawrzynska and Sirko (2016) showing higher upregulation of *SULTR1;1* in the *ein3-1* mutant than in the WT Columbia.

EIN3/EIL1 could also play a role in the crosstalk between Fe and P deficiencies related to *PHT1;5* expression. This gene, activated by the WRKY75 TF (Nagarajan et al., 2011), was not upregulated under Fe deficiency in the *ein3eil1* mutant while it was in the WT Columbia (Figure 4). It should be noted that *WRKY75* expression can be activated by ET through the EIN3/EIL1 TFs (Figure 12; Guo et al., 2017).

In conclusion, the results obtained in this work further support the existence of crosstalk between Fe, P, and S deficiency responses. In general, the responses are induced more intensively and less transiently under the specific deficiency. However, there are some exceptions, like PA induction under Fe deficiency, and *SLIM1/EIL3* upregulation under P or Fe deficiency. The results also support a relevant role for ET, through its signaling components, in the regulation of the physiological responses to the three deficiencies and in the crosstalk between them. At first, the ET constitutive *ctr1* mutant does not present constitutive activation of any of the responses while several responses are impaired in the ET insensitive *ein2-1* mutant, either under specific or nonspecific deficiencies. This suggests an important role for EIN2 in the activation and crosstalk of several nutrient deficiency responses. However, in the ET-insensitive *ein3eil1* mutant, several P and Fe deficiency responses attained their highest values of induction. These results are somewhat surprising since EIN3/EIL1 have been implicated in the activation of the key TFs FIT and PHR1 controlling Fe and P acquisition genes. At first, it would suggest the existence of additional EIN3/EIL1-independent pathways for the control of *FIT* and *PHR1* expression.

The results presented in this work along with previous published results unravel the great complexity of ET signaling in the control of nutrient deficiency responses. This complexity becomes even greater if we consider that some ET- and nutrient-related TFs, like EIN3, SLIM1/EIL3, FIT, or PHR1, could promote ET biosynthesis in an autocatalytic manner, as occurs during the ripening of climacteric fruits (Lü et al., 2018). The above TFs have been implicated in the activation of ET synthesis genes, like *MTK, SAM, ACS*, and *ACO* (Nagarajan and Smith, 2012; Lucena et al., 2015; Song and Liu, 2015; Liu et al., 2019), and consequently could promote ET synthesis. This perhaps is necessary to keep a consistent ET production along the time of the deficiency.

## Data Availability Statement

The original contributions presented in the study are included in the article/supplementary materials, further inquiries can be directed to the corresponding author/s.

## Author Contributions

FR and MG designed the experiments after discussions with RP-V and EA. MG, MA, CG, and CL conducted the laboratory work. FR, MG, RP-V, and EA wrote the manuscript that was improved by the other authors. All authors contributed to the article and approved the submitted version.

## Conflict of Interest

The authors declare that the research was conducted in the absence of any commercial or financial relationships that could be construed as a potential conflict of interest.
